# Clinical study of dorsal ulnar artery flap in hand reconstruction

**DOI:** 10.4103/0970-0358.53012

**Published:** 2009

**Authors:** Manal M. Khan, Mohd. Yaseen, L. M. Bariar, Sheeraz M. Khan

**Affiliations:** Department of Plastic Surgery, Aligarh Muslim University, J. N. Medical College, AMU, Aligarh, UP - 202 002, India

**Keywords:** Dorsal ulnar artery flap, Hand reconstruction, Island flap

## Abstract

Soft tissue defects of hand with exposed tendons, joints, nerves and bone represent a challenge to plastic surgeons. Such defects necessitate early flap coverage to protect underlying vital structures, preserve hand functions and to allow for early rehabilitation. Becker and Gilbert described flap based on the dorsal branch of the ulnar artery for defects around the wrist. We evaluated the use of a dorsal ulnar artery island flap in patients with soft tissue defects of hand. Twelve patients of soft tissue defects of hand underwent dorsal ulnar artery island flap between August 2006 and May 2008. In 10 male and 2 female patients this flap was used to reconstruct defects of the palm, dorsum of hand and first web space. Ten flaps survived completely. Marginal necrosis occurred in two flaps. In one patient suturing was required after debridement and in other patient wound healed by secondary intention. The final outcome was satisfactory. Donor areas which were skin grafted, healed with acceptable cosmetic results. The dorsal ulnar artery island flap is convenient, reliable, and easy to manage and is a single-stage technique for reconstructing soft tissue defects of the palm, dorsum of hand and first web space. Donor site morbidity is minimal, either closed primarily or covered with split thickness skin graft.

## INTRODUCTION

Providing adequate and durable coverage to obtain a healed wound with minimal scarring is a fundamental principal in the management of soft tissue defects of hands.

The soft tissue reconstruction of the hand poses a formidable challenge to the plastic and reconstructive surgeons. Durable and stable coverage of soft tissue defects of hands with a cutaneous flap seems to be an ideal solution. The paucity of local or regional flaps that are thin, pliable, and large enough, forces the surgeon to perform incomplete excisions and use skin grafts with their inherent drawbacks and suboptimal outcomes.

The dorsal ulnar artery fasciocutaneous flap was first described by Becker and Gilbert in 1988, perfused by the ascending branch of the dorsal ulnar artery, one of the major branches of the ulnar artery in the distal forearm.[1–4] The importance of this flap lies in the possibility of mobilization of tissue for reconstruction of the hand without losing a major vascular axis. The dorsal ulnar artery fasciocutaneous flap can be raised as a hinge (peninsular), or as a true island flap. In the present study, we evaluated the applications of “dorsal ulnar artery island flap” in reconstruction of soft tissue defects of hand.

## MATERIAL AND METHODS

### Patients

Twelve patients having soft tissue defects of hand were selected for coverage by dorsal ulnar artery flap between August 2006 and May 2008. Ten patients were males and two were females. They were treated with dorsal ulnar artery island flap for various defects of hand. The soft tissue defects in our study which needed dorsal ulnar artery island flap coverage were in the palm (six cases), dorsum of hand (four cases), and first web space (two cases). Patients with joint stiffness were not included in the study. Age ranged from 14 to 56 years and mean age was 32 years.

### Operative technique

Dorsal ulnar artery was localized with the help of Doppler examination [Figure 1a]. Operation was done under general anaesthesia or axillary block with tourniquet control and loupe magnification. Soft tissue defect of hand was created after scar excision in case of post-burn contracture or debridement in case of traumatic wound, and flap was designed accordingly.

**Figure 1a:**
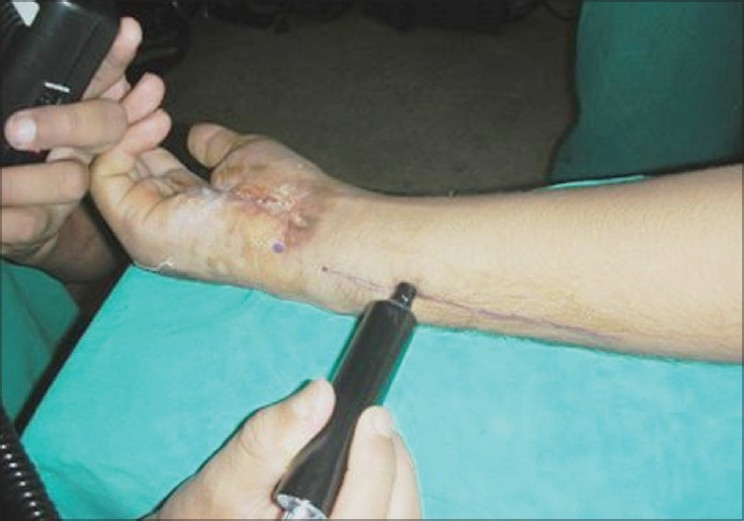
Localization of dorsal ulnar artery with doppler

The flap was raised as an island flap. The dissection started from the ulnar side of the wrist and forearm from proximal to distal including the deep fascia. The pedicle was exposed by retracting the flexor carpi ulnaris radially. The pedicle emerged from the ulnar artery 2 to 5cm proximal to the pisiform [Figures 1b and 1c]. Care was taken to preserve the dorsal branch of the ulnar nerve. The dorsal ulnar artery was dissected at its origin from the ulnar artery which permitted 180^°^ rotation of the flap. The space between the defect and the pedicle of the flap was incised, and a sulcus was created by excision of subcutaneous tissue. Tourniquet was then released, hemostasis was achieved and the flap was transferred to the defect. The subcutaneous pedicle of the flap was skin grafted to avoid tension. The donor site was closed primarily in three patients after undermining the skin flaps. These were less than 5cm wide. Larger donor defects were skin grafted. Non-adherent dressing was applied and the hand was immobilized in neutral position for about a week. Active and passive physiotherapy followed and continued for a period of three months thereafter.

**Figure 1b:**
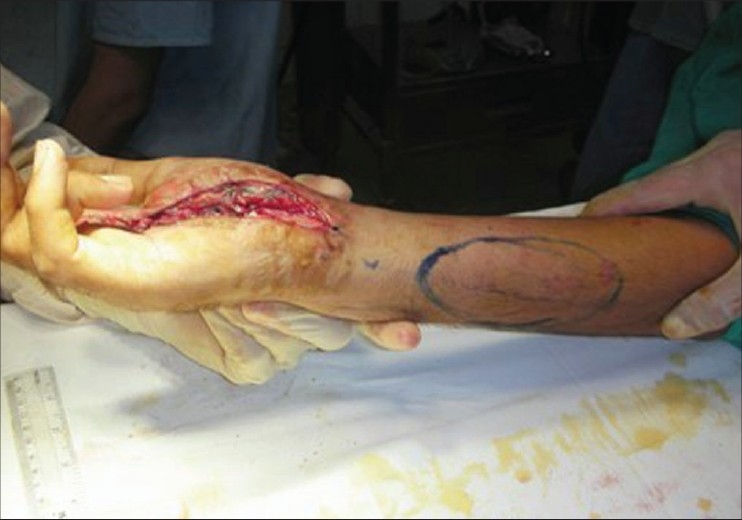
Dorsal ulnar artery flap markings

**Figure 1c:**
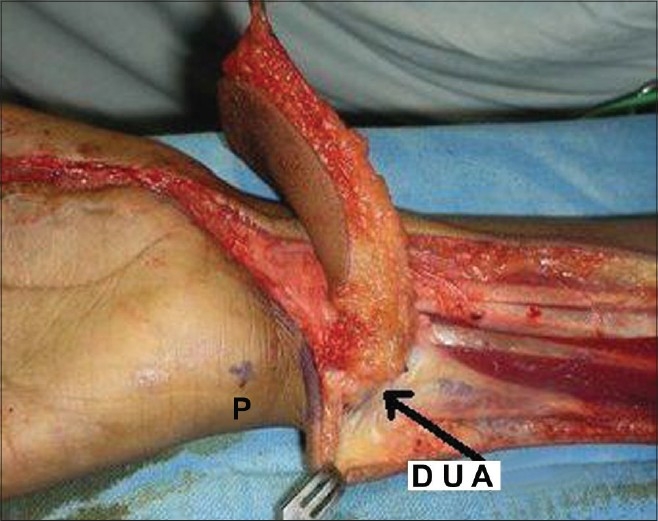
Flap pedicle showing dorsal ulnar artery

## RESULTS

All operations were successful and most patients were satisfied with the functional and cosmetic results [Figures 2a–d,3a–f and 4a–c]. The detailed data about the cases is presented in Table 1.

**Figure 2a:**
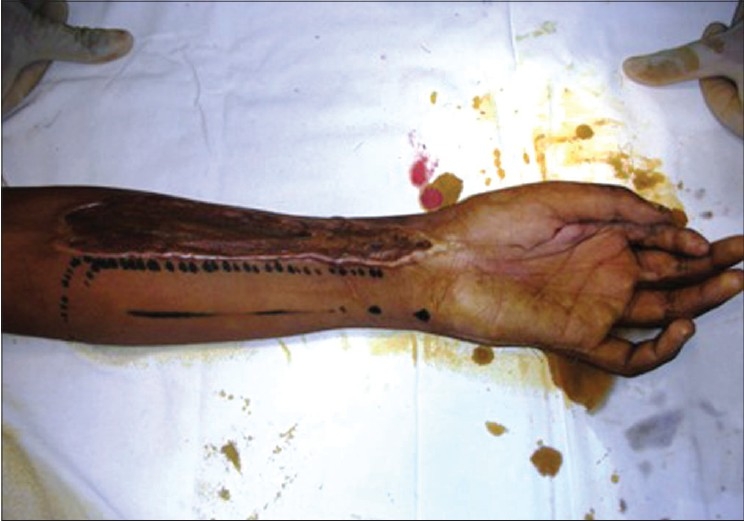
Case 1, pre-op 1^st^ web space contracture with scar forearm and planning of dorsal ulnar artery island flap

**Figure 2b:**
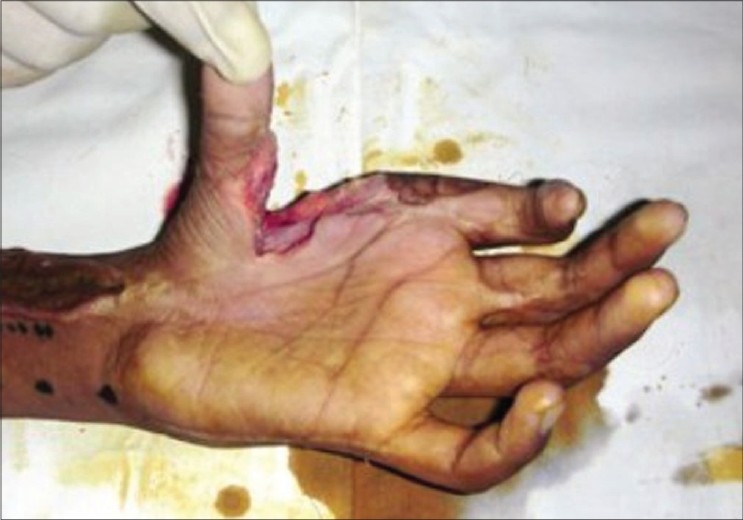
Case 1, pre-op 1^st^ web space after release of contracture

**Figure 2c:**
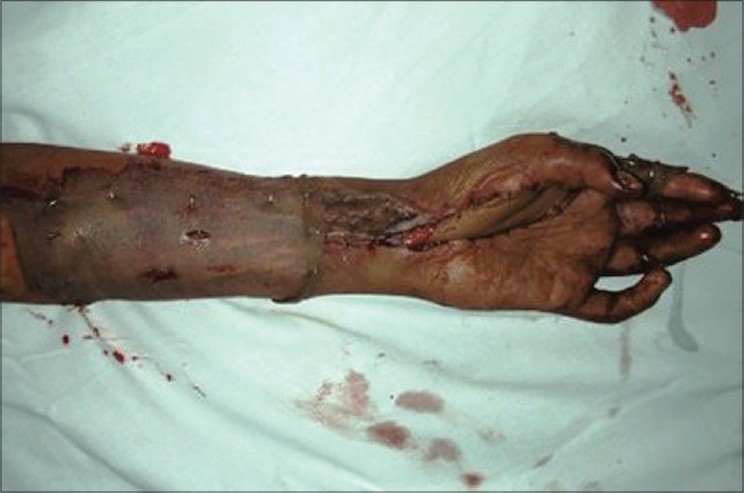
Case 1, immediate post-op dorsal ulnar artery island flap coverage of 1^st^ web space defect with skin grafted over donor area and previous scar area

**Figure 2d:**
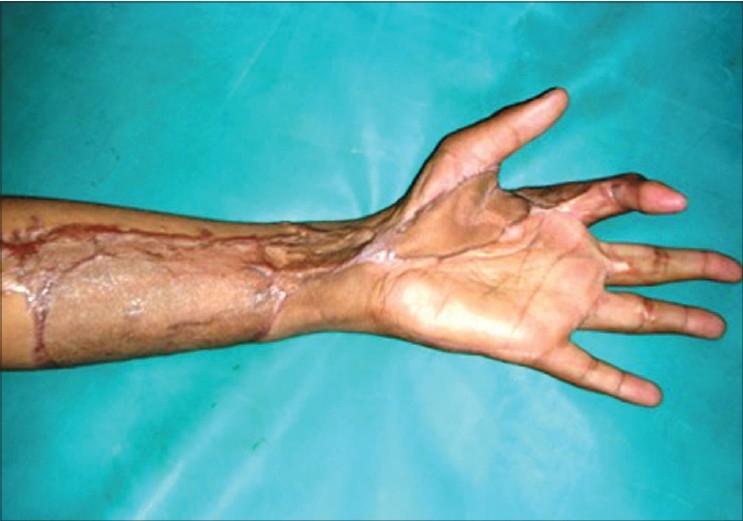
Case 1, post-op at four weeks showing good cosmetic appearance of donor area and acceptable functional results

**Figure 3a:**
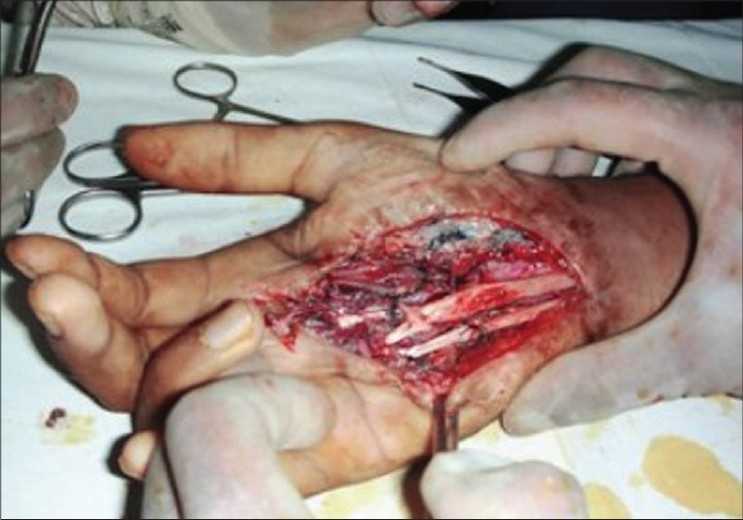
Case 2 (same case as shown in Figures 1a and b), defect of palm with exposed repaired tendons

**Figure 3b:**
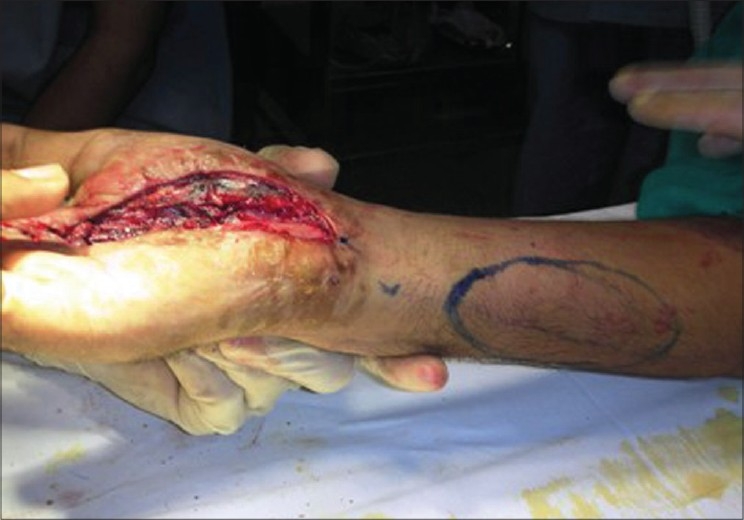
Case 2, dorsal ulnar artery island flap markings

**Figure 3c:**
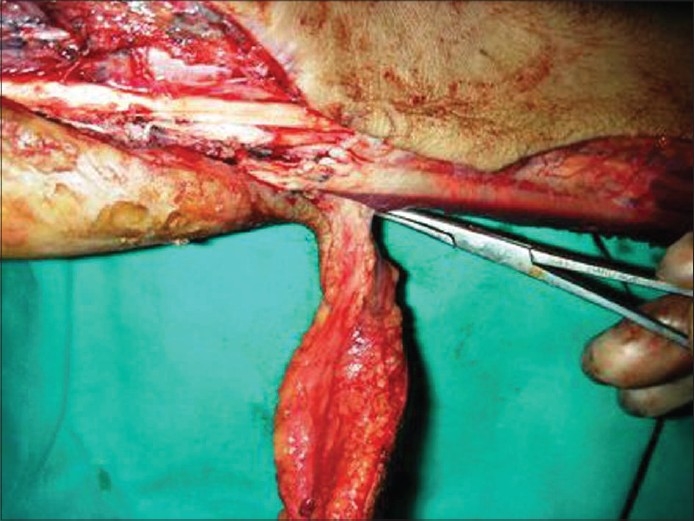
Case 2, intra-op, after raising of dorsal ulnar artery flap showing vascular pedicle

**Figure 3d:**
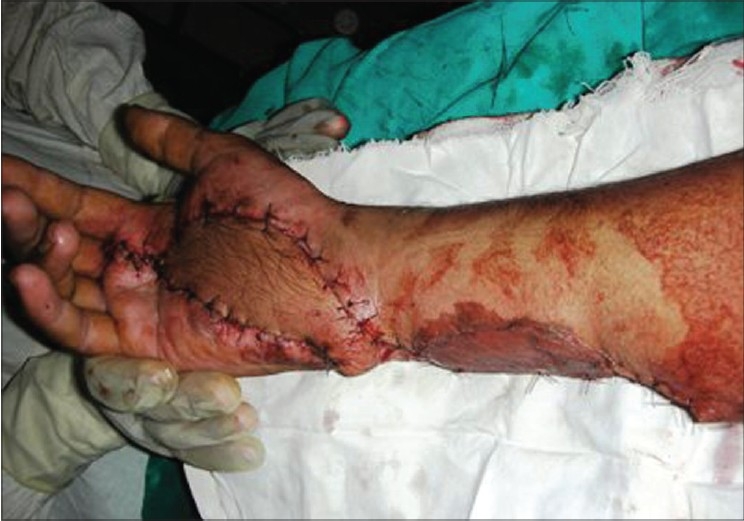
Case 2, immediate post-op dorsal ulnar artery island flap coverage of palm defect with skin grafted over donor area

**Figure 3e:**
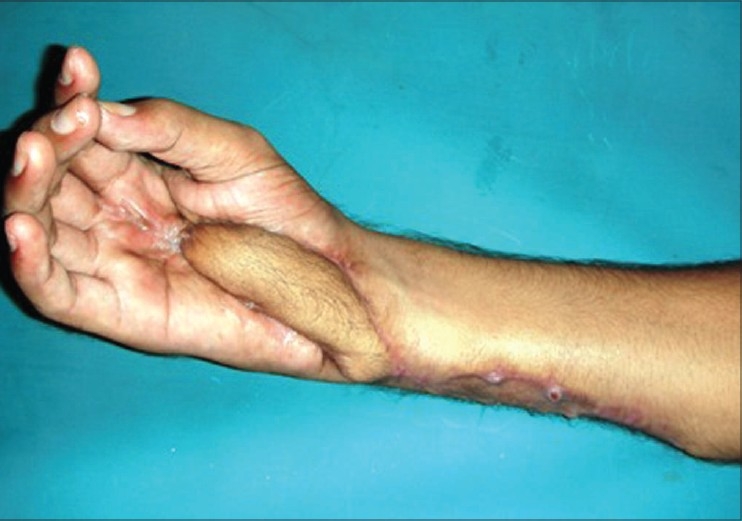
Case 2, post-op at six weeks showing good functional results and acceptable cosmetic appearance of donor area

**Figure 3f:**
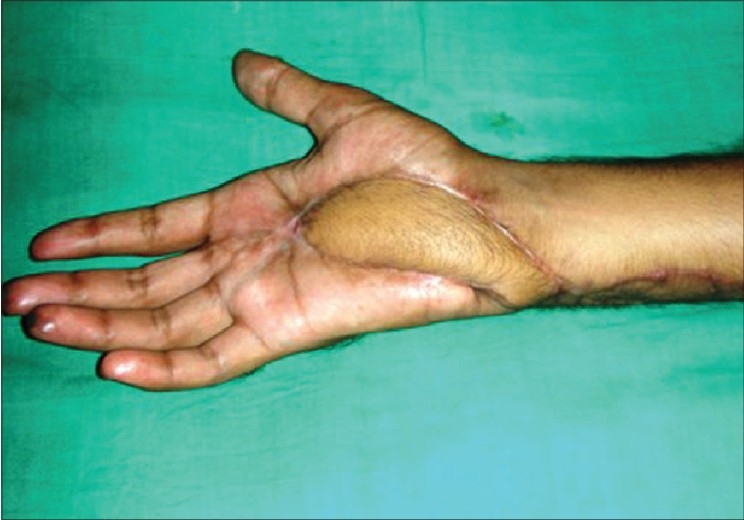
Case 2, post-op at eight weeks showing good functional results and acceptable cosmetic appearance of donor area

**Figure 4a:**
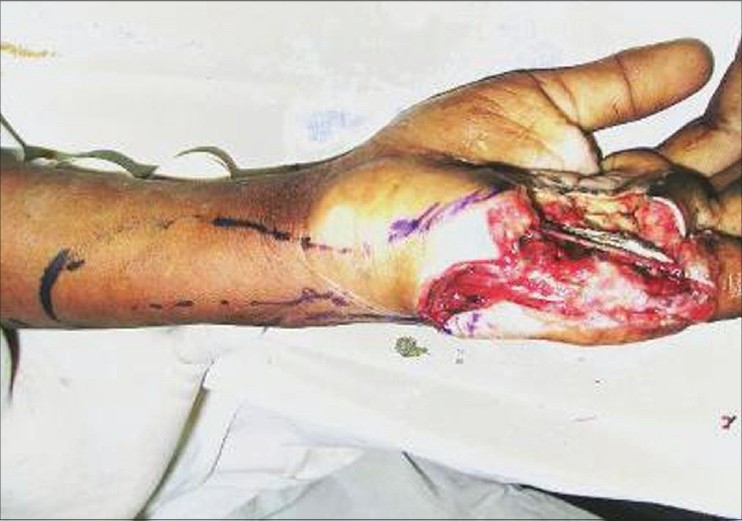
Case 3, pre-op defect of palm with dorsal ulnar artery island flap markings

**Figure 4b:**
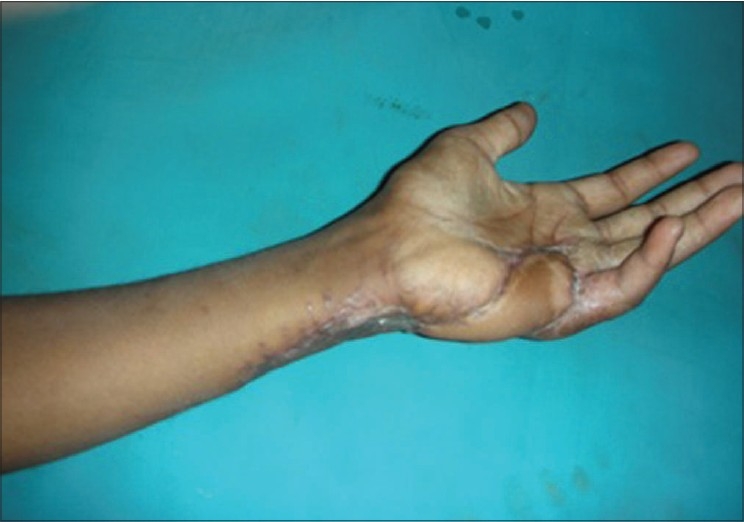
Case 3, post-op result at six weeks

**Figure 4c:**
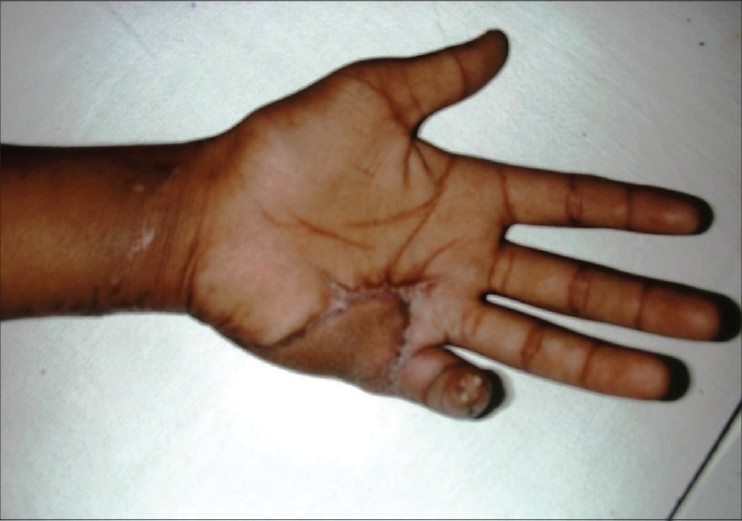
Case 3, post-op at eight weeks showing good functional and cosmetic results

**Table 1:** Patient data and summary

*Age (yrs)*	*Sex*	*Cause of tissue defect of hand*	*Post op follow-up (months)*	*Flap size (cm)*	*Donor site closure*	*Hand*	*Recipient area*
27	M	Burn contracture	6	7 × 4	STSG	Right	Palm
14	M	Burn contracture	5	8 × 3	Primary	Right	Dorsum
23	M	Post traumatic contracture	6	12 × 6	STSG	Left	Palm
34	F	Gunshot wound	6	10 × 4	STSG	Left	Palm
56	M	Burn contracture	7	12 × 5	STSG	Right	First web space
45	M	Trauma	8	7 × 3	Primary	Right	Dorsum
18	M	Post traumatic contracture	6	14 × 5	STSG	Left	First web space
32	M	Burn contracture	6	9 × 4	STSG	Left	Palm
35	M	Trauma	6	8 × 4	STSG	Right	Palm
31	F	Flame burn	5	10 × 4	STSG	Right	Dorsum
28	M	Trauma	6	10 × 5	STSG	Right	Dorsum
40	M	Trauma	6	9 × 4	Primary	Left	Palm

Complications included necrosis of distal margins of flap in two patients. Whereas in one patient suturing was required after debridement, in the second patient wound healed by secondary intention.

Donor areas which were skin grafted healed very well with acceptable cosmetic results [Figures 2d,3f and 4b].

## DISCUSSION

Soft tissue defects of hands require early coverage so that physiotherapy can be commenced as early as possible. Coverage is necessary to replace missing skin and to protect exposed structures. Various flaps have been described. The reverse radial island flap or reverse ulnar island flap have the disadvantage of sacrificing a major vascular axis. Distant flaps require long immobilization and are very discomforting. They are bulky and need thinning by yet another procedure. Free flaps require microsurgical skills and are lengthy procedures.

The dorsal ulnar artery fasciocutaneous flap was first described by Becker and Gilbert in 1988, as a local flap for covering small skin defects of hand.[1,2] The disadvantages of dorsal ulnar artery flap are the retrograde venous drainage and the relatively small defects which can be covered. Both Becker and Gilbert and Holevich-Madjarova *et al.*, have indicated that the maximum size of the dorsal ulnar artery flap should be only 10 by 5cm.[2,4] Holevich-Madjarova *et al.*, in 1991 also suggested that it should only be used for coverage of “small defects over the volar or dorsal aspect of the wrist and ulnar palm surface”. Other workers reported use of dorsal ulnar artery flap variations as the osteocutaneous flap,[6] neurocutaneous flap[3] and for different indications in hand reconstruction.[3,5,7–10] Similarly, Niranjan and Shibu in 1994 suggested that the dorsal ulnar artery flap should only be used for the ulnar side of the lower third of the forearm and the hand.[11] Antonopoulous *et al.*, in 1997 designed flaps up to the maximum theoretical limit of the territory supplied by the dorsal ulnar artery (20 cm long by 9 cm wide).[8] They used the longer length of flap to cover the defects on the radial border of the hand and wrist.

In this study, we have tried to explore the potential advantages of the dorsal ulnar artery island flap so that we can recommend it in various challenging situations for covering the soft tissue defects of hand. We have designed our flaps utilizing the territory supplied by the dorsal ulnar artery. The anterior limit of the flap could safely be placed up to the palmaris longus tendon, and posteriorly the flap may be extended to the extensor digitorum communis tendon of the fourth finger giving a width of 5 to 9 cm.[8] The longer length of the flap allowed covering the defects of the wrist, palm, radial border of the hand and the first web space. The length of the flap is determined by the major axis of the tissue loss but can extend up to 20 cm.[8]

## CONCLUSION

The dorsal ulnar artery flap is a very good local option available for the soft tissue coverage of the defects of hand without sacrificing any major vascular axis of the hand. The dorsal ulnar artery island flap is convenient, reliable, and easy to manage and is a single-stage technique for reconstructing soft tissue defects of the palm, dorsum of hand and first web space. Early use of this flap allows preservation of vital structures, decreases morbidity and allows for early rehabilitation.
